# *Schistosoma mansoni* in Susceptible and Resistant Snail Strains *Biomphalaria tenagophila*: *In Vivo* Tissue Response and *In Vitro* Hemocyte Interactions

**DOI:** 10.1371/journal.pone.0045637

**Published:** 2012-09-25

**Authors:** Rafael Nacif-Pimenta, Ana Carolina Alves de Mattos, Alessandra da Silva Orfanó, Luciene Barbosa, Paulo Filemon Paolucci Pimenta, Paulo Marcos Zech Coelho

**Affiliations:** 1 Laboratório de Entomologia Médica, Centro de Pesquisas René Rachou-Fiocruz, Belo Horizonte, Brasil; 2 Laboratório de Esquistossomose, Centro de Pesquisas René Rachou-Fiocruz, Belo Horizonte, Brasil; 3 Laboratório de Entomologia e Parasitologia Tropical - Universidade Federal de Sergipe, Aracajú, Brasil; The George Washington University Medical Center, United States of America

## Abstract

Schistosomiasis is a parasitic disease that is highly prevalent, especially in developing countries. *Biomphalaria tenagophila* is an important invertebrate host of *Schistosoma mansoni* in Brazil, with some strains (e.g. Cabo Frio) being highly susceptible to the parasite, whereas others (e.g. Taim) are completely resistant to infection. Therefore, *B. tenagophila* is an important research model for studying immune defense mechanisms against *S. mansoni*. The internal defense system (IDS) of the snail comprises hemocytes and hemolymph factors acting together to recognize self from non-self molecular patterns to eliminate the threat of infection. We performed experiments to understand the cellular defenses related to the resistance and/or susceptibility of *B. tenagophila* to *S. mansoni*. During the early stages of infection, fibrous host cells of both snail strains were arranged as a thin layer surrounding the sporocysts. However, at later stages of infection, the cellular reactions in resistant snails were increasingly more intense, with thicker layers surrounding the parasites, in contrast to susceptible strains. All parasites were damaged or destroyed inside resistant snails after 10 h of infection. By contrast, parasites inside susceptible snails appeared to be morphologically healthy. We also performed experiments using isolated hemocytes from the two strains interacting with sporocysts. Hemocyte attachment started as early as 1 h after initial infection in both strains, but the killing of sporocysts was exclusive to hemocytes from the resistant strain and was time course dependent. The resistant strain was able to kill all sporocysts. In conclusion, our study revealed important aspects of the initial process of infection related to immune defense responses of strains of *B. tenagophila* that were resistant to *S. mansoni* compared with strains that were susceptible. Such information is relevant for the survival or death of the parasites and so is important in the development of control measures against this parasite.

## Introduction

Schistosomiasis is considered to be one of the most prevalent parasitic diseases in the world. The disease occurs in 76 countries of South America, Asia and Africa. In Brazil, there are 25–30 million people living in transmission risk areas, with approximately 8 million people infected by *Schistosoma mansoni* (http://apps.who.int/tdr/svc/diseases/schistosomiasis). There are 11 species of *Biomphalaria* in Brazil [1], but only three are found to be naturally infected by *S. mansoni*: *Biomphalaria glabrata* (Say, 1818), *Biomphalaria straminea* (Dunker, 1848) and *Biomphalaria tenagophila* (d’Orbigny, 1835) [2].

*Biomphalaria tenagophila* snails are widely spread throughout Brazil, and its importance in the transmission of schistosomiasis has increased, especially in the southern and southeastern regions of the country [3]. Interestingly, different strains of the snail show different levels of resistance to *S. mansoni* infection, with some strains being highly susceptible and others completely resistant [2]. For example, *S. mansoni* is unable to elicit infection in the Taim strain of *B. tenagophila* under laboratory conditions [4]–[7]. This resistance is the result of the internal defense system (IDS) of the mollusk [8]. By contrast, the Cabo Frio strain of *B. tenagophila* is susceptible to *S. mansoni*. Consequently, *B. tenagophila* is an interesting research model in which to study IDS mechanisms against *S. mansoni*.

The parasite life cycle starts when *S. mansoni* eggs are deposited in water by an infected vertebrate host. The miracidium hatches from the egg and actively searches for its intermediate host, snails from the genus *Biomphalaria*, where upon it penetrates the tegument of the snail, preferentially targeting near the tentacles. During penetration of the tegument and once within the tissue of the snail, morphological and physiological changes occur in the miracidium and eventually it becomes a primary sporocyst. Two weeks after penetration, the primary sporocysts give birth to secondary sporocysts and these secondary sporocysts migrate to the hepatopancreas (digestive glands) and reproductive organs. Approximately 30 days after this migration, the snails start to shed cercariae into the water, which is the form of the parasite that is infective for the vertebrate host.

**Figure 1:**
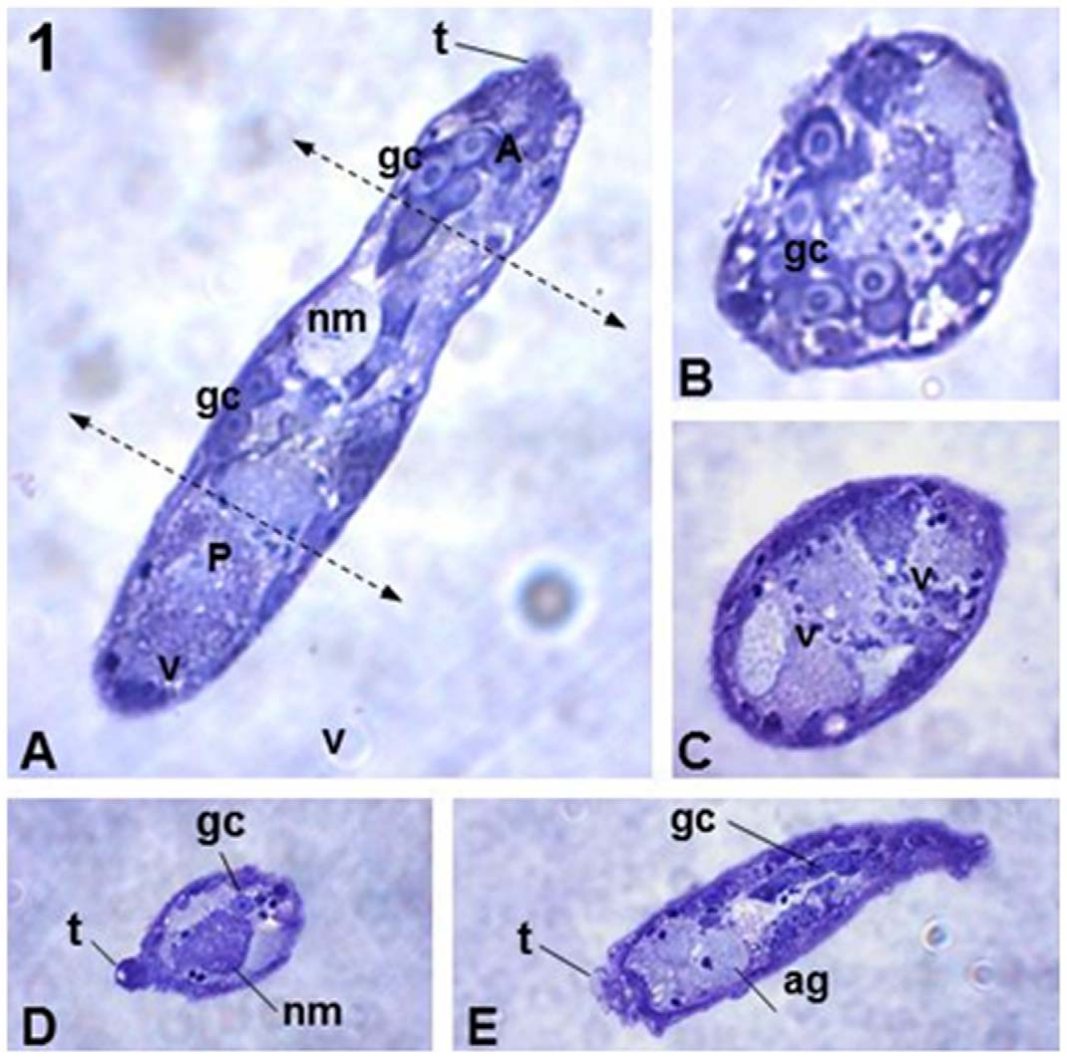
Histological sections showing distinct views of *S. mansoni* sporocysts. (**A**) Longitudinal section of the sporocyst medial plane showing the main internal organelles: germinal cells (gc), neural mass (nm), terebratorium (t) and several vesicles (v). The two dotted lines delimit the anterior (A) and posterior (P) regions of the parasite. (**B**) Transversal section of the anterior region of the parasite showing the gc. (**C**) Transversal section of the posterior region of the parasite, showing several types of v. (**D**) Oblique section of the anterior region of the parasite, showing the t, gc and nm. (**E**) Oblique sequential section of the anterior region of the parasite showing the large area of the t, as well as the apical glands (ag) and gc. Magnification 40X.

The compatibility between *S. mansoni* and *Biomphalaria* in disease transmission is determined by several genetic and physiological factors of the snail and parasite during their interaction, with the IDS that determines vector compatibility being the most important [9]. The concept of compatibility between snails and trematode worms, such as *Schistosoma*, is defined by the interaction of physiological properties of both organisms, the result of which enables the parasite to penetrate, develop and propagate inside the invertebrate host [10]. Unlike the vertebrate immune system, snails do not have lymphocytes, specific antigens immunoglobulin, or a complement system; nevertheless, they do have the capacity to recognize self from non-self [11]. The snail IDS comprises cells (hemocytes) and soluble factors contained in the hemolymph. These two components act together to determine self from non-self molecular patterns to eliminate any threats [12].

In this study, we performed *in vivo* and *in vitro* experiments to better understand the cellular immune defenses related to the resistance and/or susceptibility of the *B. tenagophila* strains, focusing on the initial phase of the interaction process between the snails and the *S. mansoni* sporocysts. This experimental model eliminates those variables encountered by using different snail species and is ideal for an increased understanding of the protective mechanisms of the invertebrate host against infection by *S. mansoni*. We used histological observation to investigate the initial tissue reactions in the two host strains in the response of the IDS to sporocyst penetration, and observed whether the parasites survived or died. We also applied laser scanning confocal microscopy (LSCM) to analyze the *in vitro* interaction between *S. mansoni* sporocysts with isolated hemocytes from the resistant and susceptible strains.

## Materials and Methods

### Snails

Specimens of *B. tenagophila* Taim and Cabo Frio strains were maintained in the Mollusk Room ‘Lobato Paraense’ of the Centro de Pesquisa René Rachou at Fiocruz in Belo Horizonte, Minas Gerais, Brazil. The *B. tenagophila* Taim strain, which was originally collected at the Ecological Station of Taim, Rio Grande do Sul, Brazil and maintained in laboratory conditions, has been completely resistant to experimental infection with *S. mansoni* [4], [13]. This strain was collected by members of our group, during the 1970s, with permission from the director of the Ecological Station of Taim at that time. The *B. tenagophila* Cabo Frio strain was initially collected in Cabo Frio, Rio de Janeiro, Brazil and showed an infection rate by *S. mansoni* of approximately 50%. This strain was also collected by members of our group, during the 1970s, following permission from the Health Department of Cabo Frio that determined the area for collection. Thus, both strains have been maintained in the laboratory for more than 25 years following field collection. All the snails were reared at 27°C in aquaria with running water, calcium carbonate substrate, artificial light and were fed daily with lettuce and ration. Only adult snails with shell diameters of 8–14 mm were used in the experiments.

**Figure 2:**
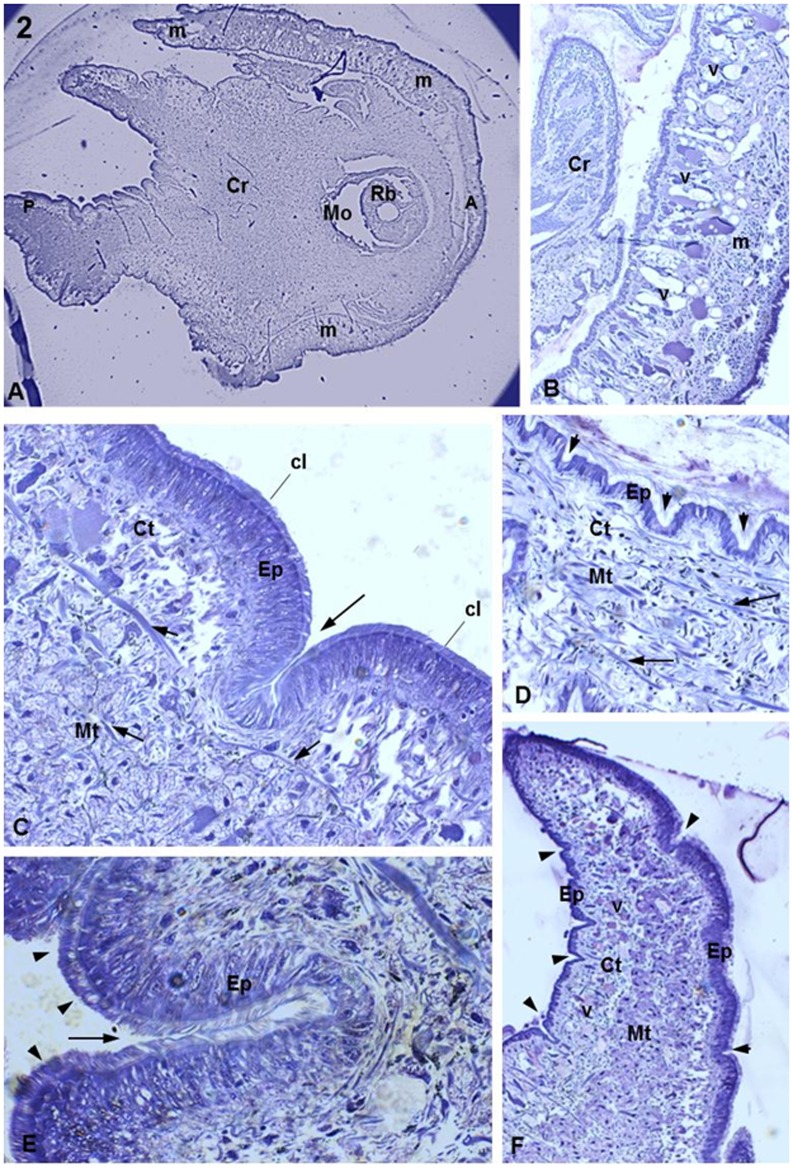
Histological sections of uninfected host *B. tenagophila* (control). (**A**) General view of the heat-foot region of *B. tenagophila*. Observe the mouth (Mo) near the anterior region (A), posterior region (P) and the mantle (m) that covers the heat-foot region (Cr). Magnification 2.5X. (**B**) Details of the region between the cr and the m. The lateral m region shows several types of vesicle (v) with distinct stain densities. Magnification 40X. (**C**) Details of the heat-foot region. Observe the ciliar cylindrical epithelium (Ep) and just below it the dense connective tissue (Ct) that is supported by the muscular connective tissue (Mt) comprising a network of muscle fibers (white arrow). A large crypt is seen that is similar to a large epithelial fold (large arrow). Small arrows indicate the cilia. Magnification 100X. (**D**) Detail of the heat-foot region, showing the Ep with several crypts (arrows) and moat-like invaginations. Key: Ct, connective tissue; Mt, muscle tissue; arrows, muscle fibers; arrowheads, crypts. Magnification: 40X. (**E**) Large magnification showing details of one crypt (large arrow) deep into the Ep. Details of the cilia on the epithelium are shown (arrowheads) Magnification: 100X. (**F**) Posterior area of the heat-foot region. Notice the presence of numerous v in the Ct supported by Mt. The Ep shows several crypts (arrowheads) Magnification 40X.

### Parasite Maintenance and Miracidium Collection

We used an LE *S. mansoni* strain maintained routinely in the Mollusk Room ‘Lobato Paraense’ of the Centro de Pesquisa René Rachou, as described elsewhere [14]. Miracidia were obtained from the liver of infected hamsters under axenic conditions and isolated using the procedures described by Chaia [15]. Briefly, the hamsters were sacrificed, the livers dissected, ground in saline solution and washed in dechlorinated water. The water was maintained in the dark for 30 min to enable egg sedimentation. The eggs were then transferred to a volumetric flask and exposed to light to stimulate egg hatching, with subsequent miracidium release and collection.

**Figure 3:**
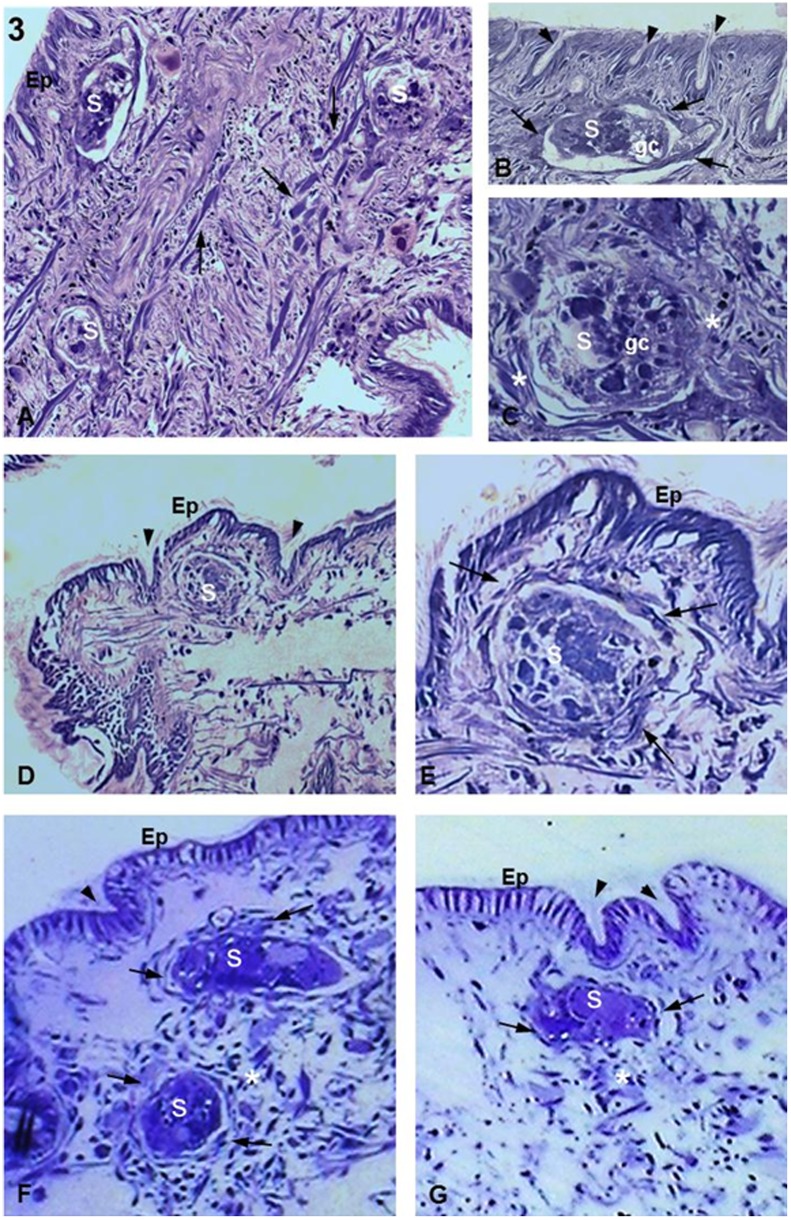
*Biomphalaria tenagophila* Cabo Frio strain infected with *S. mansoni.* (**A**) 1 h after infection. Three sporocysts (S) are seen sectioned in distinct planes, with one appearing close to the epithelial surface (asterisk). There is an absence of a tissue response or cellular infiltration. Arrows indicate muscle fibers. Magnification 20X. (**B,C**) 1 h after infection. Details of an S inside the snail tissue showing the germinal cells characteristic of the sporocyst (germ cells; gc). In (B), there is a thin layer of fibrous cells already surrounding the parasite (arrows). By contrast, (C) shows fibrous cells (asterisks) that are not forming a layer around the parasite. Arrowheads indicate crypts. Magnifications 60X. (**D**) 5 h after infection. Low magnification showing an S located below the tegument of the snail and between two crypts (arrowheads). There is no visible damage on the epithelial surface (Ep). Magnification 20X. (**E**) 5 h after infection. Details of an S near to the Ep showing a small concentration of fibrous cells (arrows) around the sporocyst forming a thin layer. Magnification 63X. (**F,G**) 10 h after infection showing two and one S, respectively, close to the Ep with no damage or signs of parasite invasion. The images also show parasites close to the epithelial crypts (arrowheads) and an unattached concentration of fibrous cells (arrows) forming thin layers around the parasites. There is a large concentration of fibrous cells in (F) comparing to (G) (asterisks). Magnification 63X.

### Snail Infection

Each snail was exposed to approximately 30 newly hatched *S. mansoni* miracidia in culture dishes for 3 hours. This number of miracidia is considered ideal for studying infection by *S. mansoni* [16] and enabled us to observe the initial interaction process of the sporocyst with the host and to find parasites easily in the snail tissues. During the study, the experimental and control snails were healthy, with no visible signs of stress or damage. The control snails survived for 8 weeks after the experiments. Infected snails were separated at set time intervals (1 h, 5 h and 10 h) and processed for histology.

**Figure 4:**
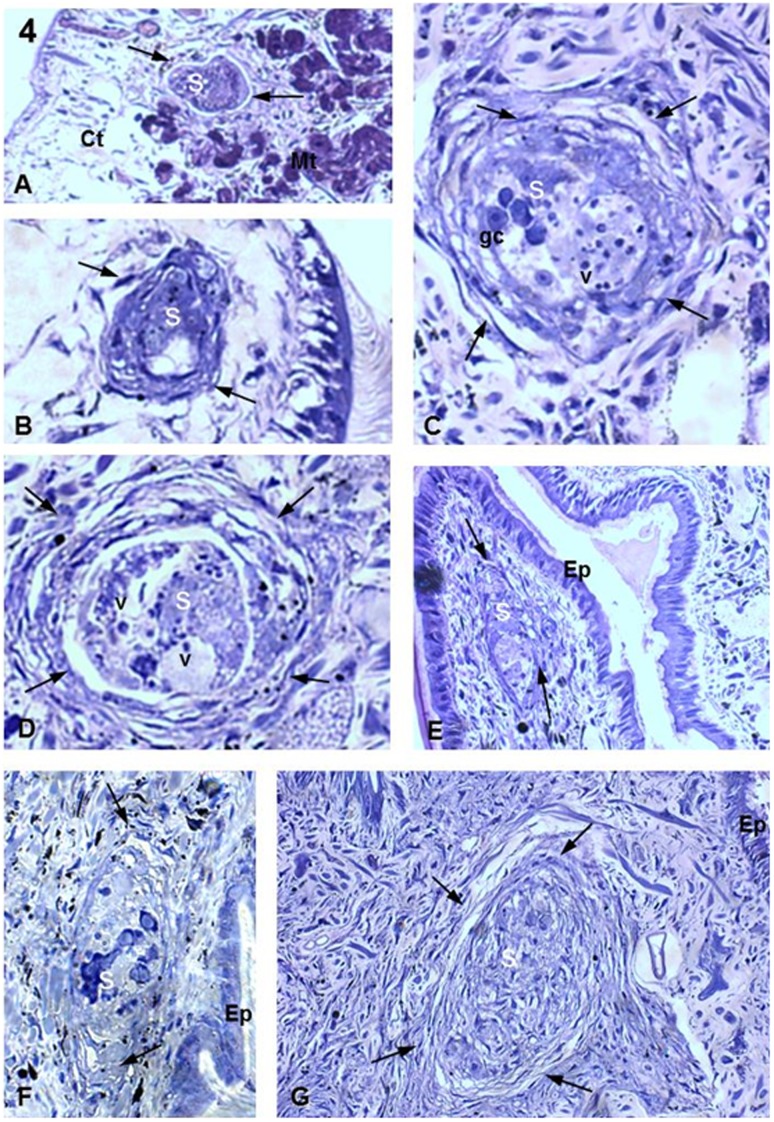
*Biomphalaria tenagophila* Taim strain infected with *S. mansoni*. (**A**) 1 h after infection, showing one sporocyst (S) between the connective (Ct) and muscular (Mt) tissues. There is already a small concentration of fibrous cells around the parasite (arrows). Magnification 20X. (**B**) 5 h after infection. Image of one S surrounded by a few fibrous cells (arrows) but already showing expressive morphological damage. Magnification 40X. (**C**,**D**) 5 h after infection. Large magnification views of S showing several dense layers of fibrous cells (arrows), indicating the strong cellular response from the snail tissue. The parasite morphology is still preserved at this stage, as granular cells (gc) and vesicles (v) can be seen. Magnification 40X. (**E**) 10 h after infection. S with little cellular response (arrows). The S is found near the area of penetration and there are no changes in the surface of the epithelium (Ep). Magnification 40X. (**F**) 10 h after infection. There is formation of a thick layer around the extremities of the parasite (arrows). The S is damaged and is located near the area of penetration of the Ep. The cellular response is not as strong as that shown in (C). Magnification 40X. (**G**) 10 h after infection. The S is completely destroyed and surrounded by several layers of fibrous cells (arrows) but remains close to the Ep. Magnification 40X.

### *In vitro* Interaction of *in vitro*-transformed *S. mansoni* Sporocysts with *B tenagophila* Hemocytes

Newly hatched miracidia obtained as described above were transformed in sporocysts as described elsewhere [17]. Briefly, the miracidia were washed, concentrated on ice in 50-mL conical polypropylene tubes and cultivated in 25-cm^2^ tissue culture flasks containing RPMI 1640 medium (Sigma-Aldrich) supplemented with 5% FBS (fetal bovine serum), and 10% penicillin/streptomycin (Sigma-Aldrich). The parasites were placed in a biochemical oxygen demand (BOD) incubator at 26°C for 24 h for miracidium differentiation. *In vitro* transformed sporocysts were washed in the medium and used immediately in the subsequent interaction experiments.

**Figure 5:**
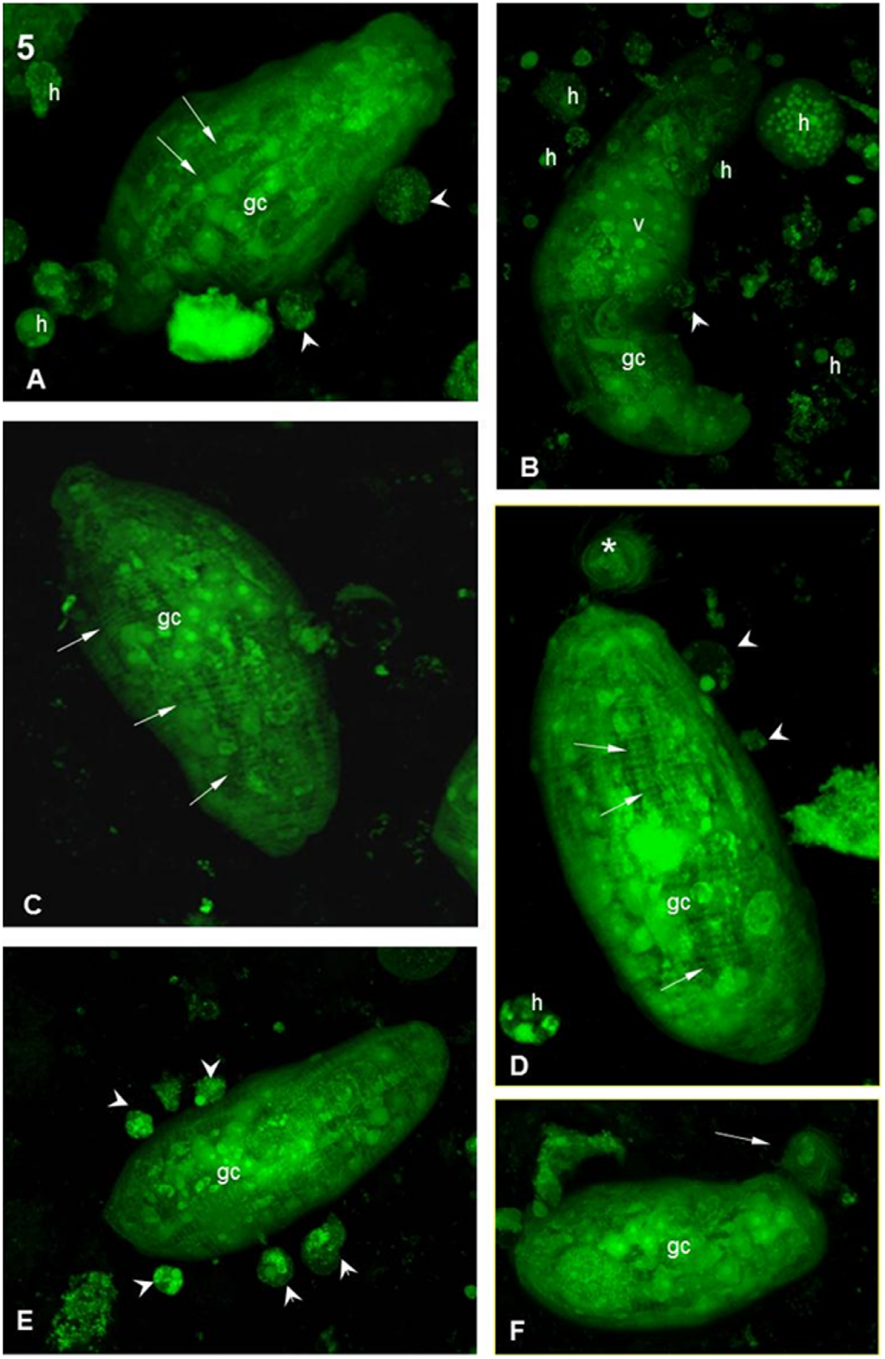
Interaction between hemocytes from the *B. tenagophila* Cabo Frio strain and *S. mansoni* sporocysts. (**A,B**) After 1 h of interaction: general appearance of a sporocyst viewed using a confocal microscope. It is possible to observe the parasite structure as well as the aspects of the hemocyte (h). There are muscle fibers surrounding the surface of the parasite (arrows), and several organelles can be seen, including germinal cells (gc) and vesicles (v). Some h are already adhered to the sporocyst (arrowheads). Magnification 63X in (A) and 40X in (B). (**C**) After 5 h of interaction, showing a sporocyst without any attached hemocytes and showing normal structures. Magnification 40X. (**D**) After 5 h of interaction showing a sporocyst with normal morphology and few small, attached hemocytes (arrowheads). There is a structure reminiscent of the ciliar plaque (asterisk). Magnification 63X. (**E**) After 10 h of interaction, showing a sporocyst with several attached hemocytes (arrowheads) on the surface without any visible damage. Magnification 40X. (**F**) After 10 h of interaction, showing a sporocyst without any attached hemocytes. There is a structure present that is reminiscent of the ciliar plaque at the extremity of the parasite (arrow). Magnification 40X.

**Figure 6:**
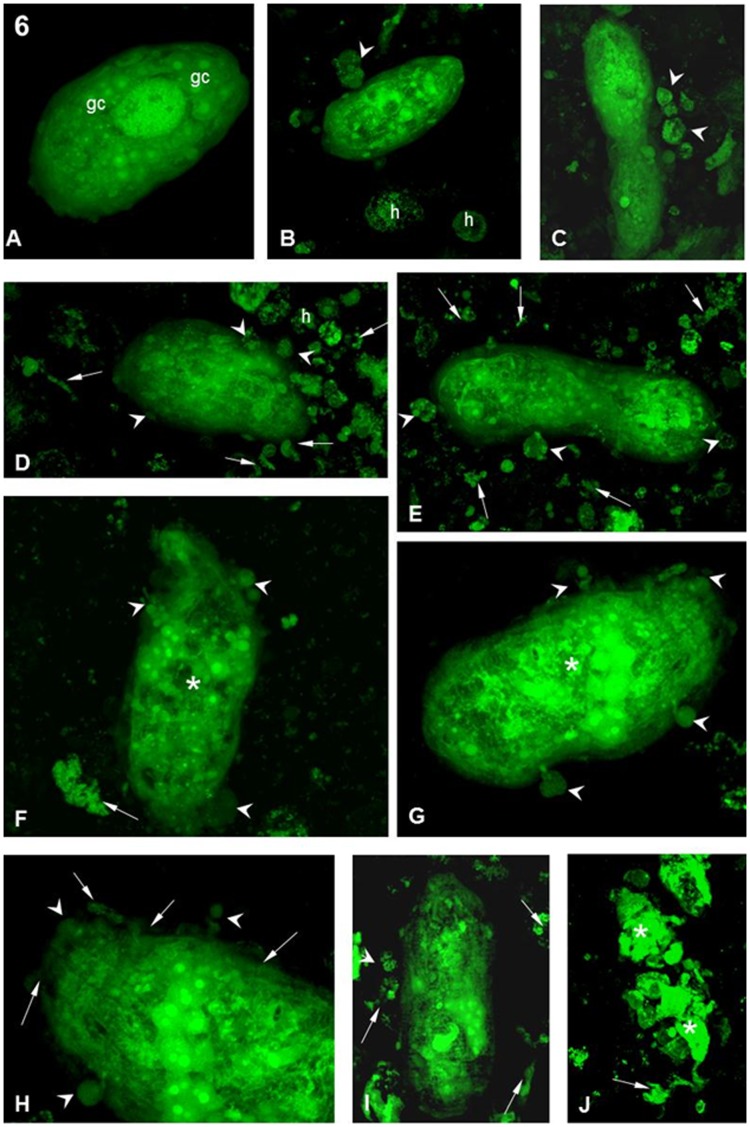
Interaction between hemocytes from the *B. tenagophila* Taim strain and *S. mansoni* sporocysts. (**A**) After 1 h of interaction, showing a sporocyst with normal morphology and no attached hemocyte. Various germinal cells are present (white arrows). Magnification 40X. (**B,C**) After 1 h of interaction, showing sporocysts with a few attached hemocytes (arrowheads). ‘h’ indicates free hemocytes. Magnification 40X. (**D,E**) After 5 h of interaction, showing porocysts with several attached (arrowheads) and free h. There is also a great quantity of cellular debris (arrows). Magnification 40X. (**F,G**) After 10 h of interaction, several attached hemocytes (arrowheads) are seen on the sporocyst. The parasite morphology is showing disturbance of the internal organelles (asterisks). Cellular debris can also be seen (arrows). Magnification 40X. (**H**) After 10 h of interaction detailing the anterior region of the sporocyst. There is a hemocyte adhered to the surface (arrowheads) and the disarrangement of the syncytial layer on the surface of the parasite. Magnification 63X. (**I**,**J**) After 10 h of interaction. sporocysts with a few attached hemocytes (arrowheads) can be seen, as well as a large amount of cellular debris around the parasite (arrows). The sporocyst in (J) is completely destroyed and is now in several parts (asterisks). Magnification 40X.

Concomitantly, *B. tenagophila* amebocyte-producing organs (APO) from the Cabo Frio and Taim strains were dissected and cultivated as described elsewhere [18]. Briefly, APO were dissected and sliced into small pieces, placed in 24-well culture plates containing CMRL 1415 medium (supplemented 10% FBS plus 4 mg/mL of fungizon and 10 mg/mL of gentamicin) and incubated for 24 h at 15°C to release hemocytes (details in [19]). Purified hemocytes from the two strains and recently transformed sporocysts were placed in 24-well culture plates to a final volume of 200 µL and allowed to interact at a proportion of 1.10^5^ hemocytes to approximately 30 sporocysts. Samples were collected at 1 h, 5 h and 10 h time intervals and glutaraldehyde fixed for analysis under LSCM.

### Histology

Experimentally infected Taim and Cabo Frio snails collected at distinct time intervals were fixed with Bouin’s solution for at least 10 h. After fixation, the soft bodies of the snails were exposed by carefully removing the hard shells using small scissors and forceps. The samples were dehydrated with increasing concentrations of ethanol (30–100%) for 30 min each, followed by embedding in historesin (Leica, Microsystems Nussloch/Heidelberg) overnight and polymerization at room temperature as described by the product protocol. Thin slices of 1 µm were sectioned randomly at different angles using a histological microtome (Micron HM 340 E) (Walldorf, Germany) and placed over warmed glass slides for staining with 1% toluidine blue [20]. The historesin resin enables 1-µm thick sections to be obtained instead of the usual 5-µm paraffin sections, providing better tissue detail and definition. The toluidine blue dye stains nucleic acids blue (a orthochromatic color) but when dye molecules bind to sulfate groups usually found in vertebrate and invertebrate tissue, the color changes from blue to purple (the metachromatic color). The stained samples were examined for the presence of *S. mansoni* and photographed during optical microscopy. In addition, to better recognize the structure of the parasites inside the snails, isolated sporocysts were embedded in 20 µl of natural commercial gelatin (unpublished method) and fixed with Karnovsky’s fixative as described below. The gelatin blocks containing the sporocysts were histological processed as described above for the snail bodies, and the stained sections were also observed under optical microscopy.

### LSCM

Samples obtained at set times during the interaction of *S. mansoni* sporocysts with hemocytes from the two strains of *B. tenagophila* were processed for observation under LSCM. Briefly, samples of interacting cells were removed from the culture plates and fixed with Karnovsky’s fixative (2.5% glutaraldehyde plus 2.5% formaldehyde in 01. M caccodylate buffer) at a pH of 7.2 for 2 hours. This method makes use of the glutaraldehyde-induced autofluorescence of proteins after cross-linking with fixative for the analysis of the cellular and subcellular structures [21]. The samples were washed in PBS (Phosphate Buffer Saline) and placed in a special device (small metal container with glass in the bottom) for observation and analyzed under the LSCM (Zeiss LSM 510) with 488-µm laser wavelength to capture the glutaraldehyde-induced green fluorescence emitted by the fixed cells. The use of the LSCM also enabled us to make three-dimensional (3D) reconstruction images from confocal sections of the process of hemocytes interacting with sporocysts, revealing not only the cells, but also details of their subcellular structures. Images were captured with a digital camera coupled to the microscope and processed for acquiring 3D images with a specific Zeiss software program (LSM 510).

### Ethical Statement

The experiments were approved by the FIOCRUZ Committee of Ethics for the Use of Animals (CEUA) (approved license number L118/09). The CEUA follows the rules established by the National Council for Control of Animal Experimentation (CONCEA). No specific permits were required for the studies using snails.

## Results

### Comparative Histology of *S. mansoni* Infection of Cabo Frio and Taim Strains of *B. tenagophila* Snails

Preliminary analysis of histological sections of isolated sporocysts embedded in gelatin enabled us to determine distinct morphologies of *S. mansoni* sporocysts within the body of the snails (Fig. 1). The parasites were elongate and approximately 70 µm in length and 20 µm in diameter. All the structures characteristic of sporocysts were recognized, including germinal cells, neural masses, small terebratorium, apical glands and several typical vesicles (Fig. 1A–E).

Histological sections of non-infected snails (control snails) enabled us to observe details of the main area of the heat-foot region, which is the area usually invaded by sporocysts. Low magnification showed the mantle that surrounds the heat-foot region of the snail (Fig. 2A). High magnification showed the internal membrane covering the heat-foot region and several vesicles stained with different intensities (Fig. 2B). The heat-foot surface was covered by a layer of ciliated cylindrical epithelial cells (Fig. 2C) forming an epithelium that was supported by dense connective tissue that in turn was supported by muscular connective tissue (Fig. 2C). Along the surface of the snail body, there were several epithelial crypts (Fig. 2D) of different sizes and shapes (Fig. 2E) inserted within the dense connective tissue. The posterior heat-foot region was characterized by the presence of several vesicles with stained contents (Fig. 2F).

After the experimental infections, histological sections showed that the two strains of *B. tenagophila* were both infected by *S. mansoni*. During the time intervals observed in this study, sporocysts were always seen in the dense and muscular connective tissues near the surface of the parasite (Fig. 3A–G).

In the *B. tenagophila* Cabo Frio strain at 1 h after infection (a.i.), the sporocysts were seen surrounded by a few fibrous cells (Fig. 3A–C). At 5 h a.i., it was also possible to observe sporocysts lodged between the epithelial crypts (Fig. 3D,E). High magnification showed details of a fine layer of fibrous cells aligned around the parasite (Fig. 3E). At 10 h a.i., the sporocysts were seen with similar features as those seen at previous time points (Fig. 3F). All the sporocysts examined had a normal morphology, with the exception of one parasite occasionally observed at 1 h a.i. (Fig. 3B). In addition, all infected snails showed intact surfaces without any visible epithelial damage resulting from parasite penetration (Fig. 3A–G).

The *Biomphalaria tenagophila* Taim strain also showed sporocysts at all infection stages examined, but with stronger tissue reactions (Fig. 4A–G). At 1 h a.i., a few fibrous cells were seen surrounding the parasites, as was also seen for the Cabo Frio snails (Fig. 4A). However, at 5 h a.i., sporocysts were seen to be surrounded by several layers of fibrous cells (Fig. 4B–D). At 10 h a.i, the last time interval examined, all observed sporocysts showed morphological damage (Fig. 4E–G), including those that were still positioned close to the surface (Fig. 4E). Numerous dense layers of host fibrous cells were seen surrounding these encapsulated and damaged parasites (Fig. 4F,G), within which all characteristic structures were completely destroyed (Fig. 4G).

### LSCA of the Interaction between *S. mansoni* Sporocysts and Hemocytes from *B. tenagophila* Cabo Frio and Taim Strains

Comparative analyses by phase contrast microscopy showed the well-known morphology of the miracidium, that is, an elongated ciliated body with a small muscular constriction in the posterior region and presence of the terebratorium. The *in vitro* transformed sporocysts showed very clearly the characteristic morphology of this parasite stage, with an absence of cilia on the surface, the presence of germinal cells, apical glands and neural masses (images not shown).

The laser confocal microscopy used to capture the glutaraldehyde-induced green fluorescence emitted by the fixed cells revealed details of the interaction of *S. mansoni* sporocysts with hemocytes from both snail strains. It was possible to compare and detect differences between the interaction processes in the two strains.

First, the laser confocal images confirmed the characteristic aspects of the *Schistosoma mansoni* sporocysts (Figs. 5A and 6A). The sporocysts, 1 h after interaction with hemocytes from the Cabo Frio strain, showed normal morphological aspects with muscle fibers around the body and germinal cells (Fig. 5A). The hemocytes were easily recognized as rounded cells with several cytoplasmic granules (Fig. 5A,B). Most sporocysts were seen to be free and only a few had hemocytes attached to their surfaces (Fig. 5A,B). After 5 h of interaction, most of the sporocysts remained free (Fig. 5C) or with only a few hemocytes attached (Fig. 5D). After 10 h of interaction, the sporocysts were similar in appearance to earlier images (Fig. 5F). Thus, there were no noticeable morphological changes in the sporocysts or in the hemocytes after 10 h of interaction (Fig. 5E).

By contrast, *Schistosoma mansoni* sporocysts 1 h a.i. with hemocytes from the Taim strain were similar in appearance to those interacting with the Cabo Frio strain at the same time, most of the sporocysts were free (Fig. 6A) and only a few had hemocytes attached to their surface (Fig. 6B,C). However, at 5 h a.i, several sporocysts had hemocytes attached over their surfaces and a large amount of cellular debris was seen around the parasite (Fig. 6D,E). At 10 h a.i., the final time point of our experiment, sporocysts were seen with different numbers of hemocytes attached (Fig. 6F–J) of distinct sizes (Fig. 6H). Several morphological changes were seen in the sporocysts (Fig. 6F,G) with most of the parasites presenting abnormal morphology. Cellular remains of destroyed sporocysts could also be seen (Fig. 6J).

## Discussion

Within the *Biomphalaria* genus, there is a large diversity in the susceptibility of strains to infection by *Schistosoma* parasites. Physiological and genetic aspects of the snail hosts, as well as the genetic factors of the *S. mansoni* parasite, contribute to this diversity, as demonstrated in distinct experimental models [22]–[24]. Until now, most interaction studies have been conducted using *B. glabrata–S. mansoni* as an experimental model [11], [25]–[29]. However, recent interaction studies have been developed with another important invertebrate host, *B. tenagophila* [5]–[8], [13], [30]–[34]. This experimental model has an important advantage, because the *B. tenagophila* Taim strain is resistant whereas the Cabo Frio strain is susceptible to *S. mansoni* infection; thus, it is possible to develop comparative experimental infections with individuals from the same species.

Despite several studies on snail infection by *S. mansoni*, only a few have characterized phenomena related to the resistance versus susceptibility of the snail tegument after parasite infection. However, these studies analyzed the parasite invasion process from 10 h to12 h after infection of the snail. Such times might miss important processes because it is already known that sporocysts are completely destroyed by *B. glabrata* resistant strains 12 h after penetration [35], [36]. In addition, these *in vitro* studies examined the cellular response of the snails and demonstrated that hemocytes isolated from *B. glabrata* and *B. tenagophila* killed sporocysts at 24 h and 48 h after interaction [37]. It is certain at these late time intervals that various aspects of the resistance–susceptibility mechanisms connected to the IDS have already been established in the snail hosts. In the present study, we focused our experiments on understanding the snail defense responses that are triggered at the start of the infection process. Thus, from 1 h to 10 h after infection, we compared the histology of sporocysts interacting with hemocytes of susceptible versus resistant *B. tenagophila* strains.

Observations by Pan [25] at 48 h after infection showed that 90% of parasites penetrated and remained in the *B. glabrata* heat-foot region and only a few migrated into deeper tissues. Similarly, in our study of *B. tenagophila* infection by *S. mansoni* that focused on much earlier time observations, we noticed that sporocysts were always found near the epithelium of the heat-foot region. It appears that the parasite penetrates the snail and initiates development in the exposed heat-foot region of the *B. tenagophila*, mainly in the base of tentacle. Moreover, our data suggest that *S. mansoni* invasion of both *B. tenagophila* strains occurs preferentially throughout the epithelial crypts (moat-like invaginations of the mantle epithelium), given that parasites were frequently found near these structures. In addition, we also observed that in both strains of *B. tenagophila*, the snail tegument did not seem to suffer any notable damage resulting from penetration by the miracidia.

After penetration of the snail tissue, *S. mansoni* miracidia undergo morphological and physiological changes, and become primary sporocysts, which then go on to complete their life cycle within the intermediate host. During this process, the parasites have to interact with the IDS of the snail connective tissue, including soluble factors and hemocytes. Several authors have observed the presence of an extracellular matrix surrounding and wrapping the sporocysts after penetration of the snail [27], [38]–[40]. In our analyses, we noticed that even at the earliest time point (1 h a.i), fibrous host cells of both snail strains were arranged as a thin layer around the sporocysts. However, observation at longer time periods demonstrated that, in resistant snails, unlike in susceptible snails, the cellular reactions were increasingly more intense, with numerous fibrous cells forming thicker layers surrounding the parasites. This aspect was more evident at our last observation time (10 h a.i.), when all parasites were damaged or completely destroyed inside the resistant strain. By contrast, the parasites inside the tissue of the susceptible snails, besides having few surrounding fibrous cells, appear to be intact and morphologically healthy and viable. It appears that early on in the process of *S. mansoni* infection of *B. tenagophila* snails, the IDS responses from both susceptible and resistant strains are similar. However, after 10 h a.i., the strong defense machinery of the resistant strain is able to react and kill all the parasites.

To better understand the host reaction of *B. tenagophila* resistant and susceptible strains to *S. mansoni* in relation to cellular defenses, we performed *in vitro* interaction experiments using isolated snail hemocytes from the two strains and *in vitro* transformed sporocysts. The structural aspects of these sporocysts were confirmed by their morphology and their viability by their capacity to develop infection in snails of both strains.

According to Souza & Andrade [41], hemocytes are differentiated from the hematopoietic tissues that occur in several parts of the snails. The main function of the hemocytes is to circulate freely in the tissue and hemolymph to recognize and phagocytize non-self antigens, including invading microorganisms, such as viruses and bacteria, or to encapsulate bigger structures, such as helminthes, and then destroy them [12]. Indeed, the hemocytes produce and secrete proteolytic proteins, which are soluble hemolymph factors that opsonize and aggregate non-self antigens to facilitate the phagocytosis [42]. The snail IDS recognize and destopsonizens using soluble hemolymph factors associated with hemocytes [32], [43]. The surface of the hemocytes has several lectin ligands that are heterogeneous between species and strains [32], [33]. Distinct types of hemocyte express specific lectin receptors on their cell surface [44]. It is already known that the action of hemocytes on the snail body against the *S. mansoni* is associated with the presence and response of soluble factors from hemolymph [8], [9], [11], [13], [45]. *In vivo* studies demonstrated that, in susceptible snails, there is a weak hemocyte reaction around the sporocysts that does not interfere in the development of the parasite infection [25]. By contrast, in resistant snails, the hemocytes are able to recognize, encapsulate and destroy the parasites [9], [46].

Our *in vitro* experiments suggest that, during the interaction process, chemotactic substances and others factors that promote the encounter and attachment are probably produced by either *B. tenagophila* hemocytes or *S. mansoni* sporocysts, or both in cooperation. It appears that these factors act in a similar way in both strains because hemocytes are equally attracted and attached on the sporocysts at identical time intervals. By contrast, our experiments also showed that the hemocyte killing properties of the two strains act in different ways, given that only those from the Taim strain (the resistant snails) were able to destroy the parasites.

The hemocyte attachment in the sporocyst starts early, just 1 h after interaction, for both strains, but the parasite killing appears to be an exclusive function of the hemocytes from the resistant strain and is time course dependent. We only observed damaged parasites when they were attached to Taim hemocytes and 5 h to 10 h a.i. This could be because of the lack of killing factors released by the hemocytes that were still not activated during the early stages of the interaction process. In addition, these killing factors appear to be secreted only by hemocytes from resistant snails and are not present in those from susceptible snails. The stronger hemocyte action from the resistant strain against the sporocysts was easily observed, as the sporocysts were structurally damaged and unviable. The hemocytes produce factors that determine the intensity level of the reaction that occurs in the susceptible or resistant strains. Consequently, the cytotoxicity that occurs during the hemocyte–sporocyst interaction and causes parasite death is dependent on the hemocyte action, which is correlated with the level of resistance or susceptibility of the snail host.

There is a little information about the nature of the factors that determine encapsulation and death of *Schistosoma* sporocysts. Boswell & Bayne [47] showed that sporocysts previously treated with concanavalin A lectin become encapsulated, but do not die in snails that are usually resistant to infection. Similar to our results, Meuleman *et al.* [48] showed that the encapsulation process occurs in either resistant or susceptible strains, but only results in parasite death in the resistant snail strain. Other authors hypothesize that it is necessary to have specific recognition for encapsulation and an effective cytotoxic response. Azevedo and collaborators [49] argued that hemocytes are able to respond to a local stimuli without the immediate action of soluble factors that are located in other regions of the host, in other words, the joint actions of local cells along with the extracellular matrix together have the ability to trigger the initial defense response. Nevertheless, we observed no encapsulation around sporocysts during our *in vitro* interaction experiments, as we worked with isolated hemocytes and sporocysts; that is, we demonstrated the interaction process and parasite killing without other possible tissue factors. The presence of peroxidase enzymes around the encapsulated sporocysts has been described during interaction experiments [12]. It is possible that, during hemocyte attachment, a process of cellular activation occurs that triggers the release of peroxidase enzymes and other cytotoxic substances around the parasites. Further studies are necessary for a better understanding of the hemocyte attachment and killing processes of the sporocysts.

In conclusion, our study revealed important aspects of the IDS responses of a *B. tenagophila* resistant strain compared with those of a strain susceptible to *S. mansoni* shortly after the initial process of infection. These responses are relevant to the survival and consequent development of the infection, resulting in either the death or survival of the parasites. This knowledge establishes the basis for future studies to understand more completely the mechanisms of *Biomphalaria* IDS acting against *S. mansoni*.

## References

[1] Teodoro TM, Janotti-Passos LK, Carvalho Odos S, Caldeira RL (2010) Occurrence of *Biomphalaria* cousini (Mollusca: Gastropoda) in Brazil and its susceptibility to *Schistosomia mansoni* (Platyhelminths: Trematoda). Mol Phylogenet Evol 57: 144–51. 20580934 10.1016/j.ympev.2010.05.019

[2] Paraense WL (1970) Planorbídeos hospedeiros intermediários do *Schistosoma mansoni*, 13–20. In: AS Cunha, Esquistossomose mansoni. Sarvier & USP, São Paulo.

[3] Paraense WL, Corrêa LR (1978) Differential susceptibility of *Biomphalaria tenagophila* populations to infection with a strain of *Schistosoma mansoni* . J Parasitol 64: 822–26. 722455

[4] Santos MBL, Freitas JR, Correia MCR (1979) Suscetibilidade ao *Schistosoma mansoni* de híbridos de *Biomphalaria tengophila* to Taim, RS, Cabo Frio, RJ e Belo Horizonte, MG. Rev Inst Med Trop 21: 281–86. 550285

[5] Rosa FM, Caldeira RL, Carvalho OS, Coelho PMZ (2004) Dominant Character of the Molecular Marker of a *Biomphalaria tenagophila* strain (Mollusca: Planorbidae) resistant to *Schistosoma mansoni* . Mem Inst Oswaldo Cruz 99: 85–7. 10.1590/s0074-02762004000100015 15057353

[6] Rosa FM, Godard ALB, Azevedo V, Coelho PMZ (2005) *Biomphalaria tenagophila*: Dominant character of the resistance to *Schistosoma mansoni* in descendants of crossbreedings between resistant (Taim, RS) and susceptible (Joinville, SC) strains. Mem Inst Oswaldo Cruz 100: 19–23. 10.1590/s0074-02762005000100004 15867958

[7] Coelho PMZ, Carvalho OS, Andrade ZA, Martins-Sousa RL, Rosa FM, et al (2004) *Biomphalaria tenagophila/Schistosoma mansoni* interaction: premises for a new approach to biological control of schistosomiasis. Mem Inst Oswaldo Cruz 99: 109–111. 15486646 10.1590/s0074-02762004000900020

[8] Coelho JR, Bezerra FS (2006) Compatibility of *Biomphalaria tenagophila* with *Schistosoma mansoni*: a study of homologous plasma transference. Mem Inst Oswaldo Cruz 101: 111–12. 10.1590/s0074-02762006000100022 16699720

[9] Negrão-Corrêa D, Pereira CAJ, Rosa FM, Martins-Souza RL, Andrade ZA, et al (2007) Molluscan response to parasite: *Biomphalaria* and *Schistosoma mansoni* interaction. Invert Survival J 4: 101–11.

[10] Cheng T (1968) The compatibility and incompatibility concept as related to trematoed and molluscs. Pacific Sci. 22: 141–150.

[11] Bayne CJ, Buckley PM, DeWan PC (1980) *Schistosoma mansoni*: cytotoxicity of hemocytes from susceptible snail hosts for sporocysts in plasma from resistant *Biomphalaria glabrata* . Exp Parasitol 50: 409–16. 7428914 10.1016/0014-4894(80)90043-0

[12] Van Der Knaap WPW, Loker ES (1990) Immune mechanisms in trematode-snail interactions. Parasitol Today 6: 175–182. 15463334 10.1016/0169-4758(90)90349-9

[13] Pereira CA, Martins-Souza RL, Corrêa A Jr, Coelho PMZ, Negrão-Corrêa D (2008) Participation of cell-free haemolymph of *Biomphalaria tenagophila* in the defence mechanism against *Schistosoma mansoni* sporocysts. Parasite Immunol 30: 610–19. 19067842 10.1111/j.1365-3024.2008.01062.x

[14] Pellegrino J, Katz N (1968) *Experimental chemotherapy of Schistosomiasis mansoni* . Adv Parasitol 6: 233–90. 4978052 10.1016/s0065-308x(08)60475-3

[15] Chaia G (1968) Technique for concentration of miracidia. Rev Bras Malar Doencas Trop. 8: 355–57. 13494853

[16] Souza CP (1993) *Schistosoma mansoni*: Aspectos Quantitativos da Interação Hospedeiro-parasito e Desenvolvimento em *Biomphalaria glabrata*, *Biomphalaria tenagophila* e *Biomphalaria straminea*, PhD Thesis, ICB–UFMG, Belo Horizonte, 315 pp.

[17] Mattos AC, Kusel JR, Pimenta PF, Coelho PMZ (2006) Activity of praziquantel on in vitro transformed *Schistosoma mansoni* sporocysts. Mem Inst Oswaldo Cruz 101 (Suppl 1)283–7. 17308783 10.1590/s0074-02762006000900044

[18] Barbosa L, Silva LM, Coelho PMZ, Santos SR, Fortes-Dias CL (2006) Primary culture of the region of the amebocyte-producing organ of the snail *Biomphalaria glabrata*, the intermediate host of *Schistosoma mansoni* . Mem Inst Oswaldo Cruz 101(6): 639–43. 17072476 10.1590/s0074-02762006000600010

[19] Sullivan JT (1990) Long-term survival of heterotopic allgrafts of amebocyte-producing organ in *Biomphlaria glabrata* (Mullusca: Pulmonata). Trans Am Microsc Soc 111: 1–15.

[20] Silva LM, Botelho AC, Nacif-Pimenta R, Martins GF, Alves LC, et al (2008) Structural analysis of the venom glands of the armed spider *Phoneutria nigriventer* (Keyserling, 1891): Microanatomy, fine structure and confocal observations. Toxicon 51: 693–706. 18241905 10.1016/j.toxicon.2007.12.009

[21] Fester T, Berg RH, Taylor CG (2008) An easy method using glutaraldehyde-introduced fluorescence for the microscopic analysis of plant biotrophic interactions, J. Microscopy. 2: 342–48. 10.1111/j.1365-2818.2008.01999.x 18778431

[22] Basch PF (1979) Intermidiate host specificity in *Schistosoma mansoni*. Exp. Parasitol 39: 150–69. 10.1016/0014-4894(76)90022-9 767127

[23] Richards CS (1976) Genetics of the host-parasite relationship between *Biomphalaria glabrata* and *Schistosoma mansoni*. Genetics of the host-parasite relationship between host-parasite relationships. Symp Brithis Soc Parasitol 14: 45–54.

[24] Richards CS, Shade PC (1987) The genetic variation of compatibility in *Biomphalaria glabrata* and *Schistosoma mansoni* . J Parasitol 73: 1146–1151. 3437352

[25] Pan C (1965) Studies on the host–parasite relationship between *Schistosoma mansoni* and the snail *Australorbis glabratus* . Am J Trop Med Hyg14 6: 1965. 10.4269/ajtmh.1965.14.931 5840648

[26] Lie KJ, Jeong KH, Heyneman D (1979) Immune Responses in Parasitic Infections (Soulsby, E. J. L., ed.), 211–270 CRC Press.

[27] Bezerra FSM, Nogueira-Machado JA, Chaves MM, Correa RF, Coelho PMZ (2003) Effect of gamma radiation on the activity of hemocytes and the course of *Schistosoma mansoni* in resistant *Biomphalaria glabrata* . Mem Inst Oswaldo Cruz 98: 73–5. 12700864 10.1590/s0074-02762003000100010

[28] Spray FJ, Granath WO Jr (1988) Comparison of haemolymph proteins from *Schistosoma mansoni* (Trematoda)-susceptible and resistant *Biomphalaria glabrata* (Gastropoda). Comp Biochem Physiol 91: 619–24. 10.1016/0305-0491(88)90182-4 3224503

[29] Coustau C, Yoshino TP (1994) Surface membrane polypeptides associated with hemocytes from *Schistosoma mansoni*-susceptible and -resistant strains of *Biomphalaria glabrata* (Gastropoda) J Invertebr Pathol. 63: 82–84. 10.1006/jipa.1994.1013 8106743

[30] Bezerra FSM, Nogueira-Machado JA, Chaves MM, Martins RL, Coelho PMZ (1997) Quantification of the number and phagocytary activity of hemocytes of resistant and susceptible strains of *Biomphalaria glabrata* and *Biomphalaria tenagophila* infected with *Schistosoma mansoni* . Rev Inst Med Trop 39: 197–201. 10.1590/s0036-46651997000400003 9640781

[31] Rosa FM, Godard ALB, Azevedo V, Coelho PMZ (2005) *Biomphalaria tenagophila*: Dominant character of the resistance to *Schistosoma mansoni* in descendants of crossbreedings between resistant (Taim, RS) and susceptible (Joinville, SC) strains. Mem Inst Oswaldo Cruz 100: 19–23. 10.1590/s0074-02762005000100004 15867958

[32] Martins-Souza RL, Pereira CAJ, Martins Filho OA, Coelho PMZ, Corrêa Jr A, et al. (2006) Differential lectin labelling of circulating hemocytes from *Biomphalaria glabrata* and *Biomphalaria tenagophila* resistant or susceptible to Schistosoma mansoni infection. Mem Inst Oswaldo Cruz 101(Suppl. I): 185–192. 10.1590/s0074-02762006000900029 17308768

[33] Martins-Souza RL, Pereira CA, Rodrigues L, Araújo ES, Coelho PMZ, et al (2011) Participation of N-acetyl-D-glucosamine carbohydrate moieties in the recognition of *Schistosoma mansoni* sporocysts by haemocytes of *Biomphalaria tenagophila.* . Mem Inst Oswaldo Cruz 106: 884–91. 22124562 10.1590/s0074-02762011000700015

[34] Mattos AC, Martins-Souza RL, Kusel JR, Coelho PMZ (2011) Interaction between primary and secondary sporocysts of *Schistosoma mansoni* and the internal defence system of *Biomphalaria* resistant and susceptible to the parasite. Mem Inst Oswaldo Cruz 106: 424–32. 21739029 10.1590/s0074-02762011000400007

[35] Cousin C, Ofori K, Acholonu S, Miller A, Richards C, et al (1985) *Schistosoma mansoni*: Changes in the albumen gland of *Biomphalaria glabrata* snails selected for nonsusceptibility of the parasite. J. Parasitol 81: 905–911. 8544062

[36] Hahn UK, Bender RC, Bayne CJ (2001) Involvement of nitric oxide in killing of *Schistosoma mansoni* sporocysts by hemocytes from resistant *Biomphalaria glabrata* . J Parasitol 87: 778–85. 11534641 10.1645/0022-3395(2001)087[0778:IONOIK]2.0.CO;2

[37] Barbosa L, Caldeira RL, Carvalho OS, Vidigal TH, Jannotti-Passos LK, et al (2006) Resistance to *Schistosoma mansoni* by transplantation of APO *Biomphalaria tenagophila* . Parasite Immunol 28: 209–12. 16629706 10.1111/j.1365-3024.2006.00827.x

[38] Lemos QT (1999) Contribution to the histology of *Biomphalaria glabrata* Rev Soc Bras Med Trop. 32: 343–47. 10.1590/s0037-86821999000400002 10495661

[39] Yoshino TP (1976) The Ultrastructure of circulating hemolymph cells of the marine snail Cerithidea californica (Gastropoda: Prosobranchiata). J Morphol 150: 148–56.

[40] Krupa PL, Lewis LM, Del Vecchio P (1977) *Schistosoma haematobium* in *Bulinus guernei*: electron microscopy of hemocyte-sporocyst interactions. J Invertebr Pathol 30: 35–45. 925364 10.1016/0022-2011(77)90034-9

[41] Souza S dos S, Andrade ZA (2006) On the origin of the *Biomphalaria glabrata* hemocytes. Mem Inst Oswaldo Cruz 101 (Suppl I): 213–218. 10.1590/s0074-02762006000900033 17308772

[42] Richards CS, Renwrantz LR (1991) Two lectins on the surface of *Helix pomoatia* haemocytes: a Ca2+ dependent, GalNacspecific lectin and a Ca2+ independent, mannose 6-phosphate- specific lectin which recognizes activated homologous opsonins. J Comp Physiol 161: 43–45.

[43] Zelck UE, Becker W, Bayne CJ (1995) The plasma proteins of *Biomphalaria glabrata* in the presence and absence of *Schistosoma mansoni* . Dev Comp Immunol 19: 181–194. 8595817 10.1016/0145-305x(95)00012-i

[44] Joky A, Matricon-Gondran M, Benex J (1983) Fine structural differences in the amoebocytes of *Biomphalaria glabrata* . Dev Comp Immunol 7: 669–72.

[45] Granath WO Jr, Yoshino TP (1984) *Schistosoma mansoni*: passive transfer of resistance by serum in the vector snail, *Biomphalaria glabrata* . Exp Parasitol 58: 188–93. 6479289 10.1016/0014-4894(84)90034-1

[46] LoVerde PT, Shoulberg N, Gherson J (1984) Role of cellular and humoral components in the encapsulation response of *Biomphalaria glabrata* to *Schistosoma mansoni* sporocysts *in vitro* . Prog Clin Biol Res 157: 17–29. 6483876

[47] Boswell CA, Bayne CJ (1986) Lectin-dependent cell-mediated cytotoxicity in an invertebrate model: Con A does not act as a bridge. Immunology 57: 261–64. 3949370 PMC1453953

[48] Meuleman EA, Bayne ME, van der Knaap, WPW (1987) Developmental and Comparative Immunology (Cooper, E. L. Langlet, C. and Bierene, J., eds), 116–127, Alan Liss.

[49] Azevedo CM, Borges CC, Andrade ZA (2006) Changes induced in *Biomphalaria glabrata* (Say, 1818) following trials for artificial stimulation of its internal defense system. Mem Inst Oswaldo Cruz 1: 199–203. 10.1590/s0074-02762006000900031 17308770

